# FireStem2D – A Two-Dimensional Heat Transfer Model for Simulating Tree Stem Injury in Fires

**DOI:** 10.1371/journal.pone.0070110

**Published:** 2013-07-19

**Authors:** Efthalia K. Chatziefstratiou, Gil Bohrer, Anthony S. Bova, Ravishankar Subramanian, Renato P. M. Frasson, Amy Scherzer, Bret W. Butler, Matthew B. Dickinson

**Affiliations:** 1 Department of Civil and Environmental Engineering and Geodetic Science, The Ohio State University, Columbus, Ohio, United States of America; 2 Northern Research Station, U. S. Forest Service, Delaware, Ohio, United States of America; 3 Rocky Mountain Research Station, U. S. Forest Service, Missoula Fire Sciences Laboratory, Missoula, Montana, United States of America; Centrum Wiskunde & Informatica (CWI) & Netherlands Institute for Systems Biology, The Netherlands

## Abstract

FireStem2D, a software tool for predicting tree stem heating and injury in forest fires, is a physically-based, two-dimensional model of stem thermodynamics that results from heating at the bark surface. It builds on an earlier one-dimensional model (FireStem) and provides improved capabilities for predicting fire-induced mortality and injury before a fire occurs by resolving stem moisture loss, temperatures through the stem, degree of bark charring, and necrotic depth around the stem. We present the results of numerical parameterization and model evaluation experiments for FireStem2D that simulate laboratory stem-heating experiments of 52 tree sections from 25 trees. We also conducted a set of virtual sensitivity analysis experiments to test the effects of unevenness of heating around the stem and with aboveground height using data from two studies: a low-intensity surface fire and a more intense crown fire. The model allows for improved understanding and prediction of the effects of wildland fire on injury and mortality of trees of different species and sizes.

## Introduction

FireStem2D is an extensive update and expansion of FireStem [1], [2], a stem heating model developed for use by researchers and forest and fire managers for predicting effects of prescribed fires and wildfires. Prescribed burning is used for various purposes, such as restoration and maintenance of fire-dependent forests, grasslands, and savannas, clearing land for cultivation, and maintaining habitat for fire-dependent plants and animals [3]. Early attempts to predict stem injury from wildland fire included studies to detect the factors that have the greatest effect on the survival of trees exposed to fire [4], [5]. McCarthy and Sims [6] introduced an empirical method for estimating fire-caused tree mortality. Shirley [7] exposed tissues to heated baths to determine lethal temperatures and produced relations for injury as a function of time and temperature for a range of species. Later studies were conducted to determine the thermal properties of wood and bark, such as bark thickness, density, thermal conductivity, heat capacity, and moisture absorption and desorption [8]–[11]. The U.S. Forest Service led the development of empirical models to predict the probability of tree mortality from both stem and crown injury [12], [13]. Martin [14] showed that it would be possible to make mortality predictions by combining a one-dimensional (1-D) heat transfer model with a model of the denaturation of proteins at elevated temperatures. Advances to stem-heat injury prediction included the application of 1-D finite differencing methods [1], [2], [15]–[17]. Recently, Michaletz et al. [18] provided evidence that cavitation and deformation of the xylem under wildland fire conditions reduce water transport in stems and branches and may affect tree injury.

One-dimensional models assume that heating is uniform around the circumference of the stem. However, in reality heating is generally maximal on the side of the stem leeward to the wind. Leeside vortices entrain heat and combustion gasses that generate a standing leeward flame that maximizes heating on the leeside of the stem. This leads to deeper injury on the lee side, but may be insufficient to cause stem death, because injury does not occur around the entire stem [19]. A two-dimensional model can better simulate the effects that fire can have at different directions around the circumference of the tree. Understanding the impacts of the variation of the circumferential heat distribution on the eventual stem injury is very important, as a tree can survive as long as it is not girdled, which may be the case even where a narrow wedge, about 20% of the stem area, remains unaffected [20]. A two-dimensional model developed by Potter and Andresen [21] is driven by heat flux from solar radiation; however, this model was not developed or used to simulate stem heating from fires.

In this paper, we describe FireStem2D, a tree-stem heating model that provides two-dimensional (2-D) estimates of tissue (e.g., live bark, cambium, and wood) necrosis around the circumference of stems. We describe model parameterization and evaluation based on a set of laboratory stem-heating experiments. Finally, we explore model sensitivity to the unevenness of heating around the stem and height above ground using fire data from two studies of a low intensity surface fire and a more intense crown fire. The software along with documentation can be accessed in the following site: http://www.firelab.org/research-projects/physical-fire/126-firestem, or obtained from the supplemental material (Appendix S1) for this manuscript.

## Methods

### The FireStem2D Model

FireStem2D simulates the influence of fire as a dynamic and spatially heterogeneous heat-source around the circumference of a virtual horizontal slice of a stem. FireStem2D is a physically-based, thermodynamic, 2-D model of tree stem injury as a function of external heat forcing. It is conceptually based on the 1-D model FireStem [1], [2]. FireStem was the first numerical model for fire-induced heat transfer in tree stems to include a heat flux boundary condition. FireStem2D includes further developments of the forcing, numerical solver and water loss functions, and the extension to a 2-D domain that includes tangential heat flow. In contrast, Jones [23] extended FireStem in a 2-D domain by simulating radial heat transfer within adjacent 1-D wedges that did not communicate in the circumferential direction.

FireStem2D provides increased capability for predicting fire-induced mortality and injury before a fire occurs. By directly simulating tissue temperatures, moisture loss, and charring, it forecasts the depth and circumferential extent of injury caused by incident heat flux around a stem at a given height above ground. These data are further integrated to provide a depth of tissue necrosis around the stem through a tissue viability function.

**Figure 1:**
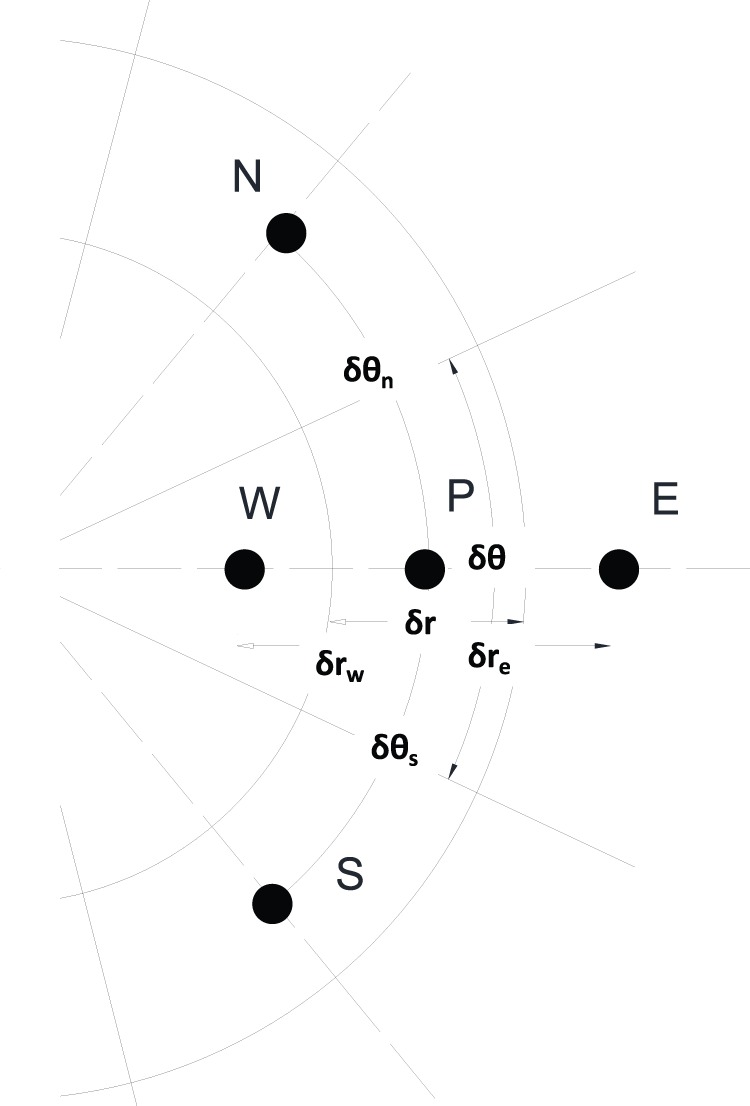
Schematic of a two-dimensional control volume used in the development of the numeric model. Stems are divided into uniform angular wedges and each wedge is discretized into nodes (e.g. *P,N,S,W,*E) in the radial direction. The distance between nodes is flexible (e.g. δr_w)_, and was set in the simulations described in this study to 1 mm. δr_e_: distance of *W* from *P*, δr_w_: distance of *E* from *P*, δθ_n_: angular distance of *N* from *P*, δθ_s_: angular distance of *S* from *P*.

Stems are simulated as circular slices (Fig. 1) [1]. The required inputs to the model include geometric information (stem diameter, outer and inner bark thickness), and physical properties (thermal conductivity, density, specific heat, moisture content in inner and outer bark, and wood). Stems are divided into uniform angular wedges and each wedge is discretized into nodes in the radial direction. The distance between nodes is flexible. The numerical solver uses a Crank-Nicolson approach [24]. Initial conditions are prescribed as a uniform temperature [21]. Conditions at the outer boundary throughout the simulation are enforced as a prescribed flux (temperature gradient). This forcing can change through time, simulating the heat flux from a fire line as it is moving past a tree. We use a periodic boundary to connect the last circumferential wedge to the first, and assume no flux boundary conditions at the inner (stem center) boundary. This assumption, taken for numerical simplicity, means that heat must diffuse around the inner-most ring of stem elements in order to cross the tree center to the opposing radial wedge. This is reasonable because the elements are very narrow at the inner-most ring and the circumference is not much longer than the distance across, and because under most realistic conditions only a marginal amount of heat reaches that depth.

### Governing Equation

The model simulates heat transfer in two dimensions. The equation uses cylindrical coordinates (*r,* for the radial axis coordinate – from bark to core in meters, and *θ*, for the circumferential axis coordinate – around the stem in radians) to describe the heat transfer radially (first part of right side of equation 1) and circumferentially (second part of right side of equation 1):
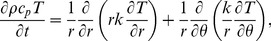
(1)where *t* is the time coordinate (seconds), *ρ* is the moist stem (wood+water) density (kg/m^3^), which is variable in time and space and calculated using empirical equations (3), and (6) (for the wood and bark, respectively), *k* is conductivity (W/m/K) calculated using empirical equations (4) and (7) (for the wood and bark, respectively), *c_p_* is heat capacity (J/kg/K) calculated using empirical equations (5) and (8) (for the wood and bark, respectively), and *T i*s temperature (K). Equation 1 is solved by numerical integration over a small, finite, annular control volume (*r_w_ : r_e_*, 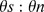, shown schematically in Fig. 1), (following [15]), and a finite incremental time step, 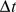, using a Crank-Nicholson time-integration scheme:
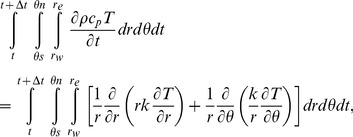
(2)


### Thermophysical Properties of Wood

The moist density of the wood and its thermal conductivity are not assumed constant in the model. Both of these thermophysical properties vary in space and time and are affected by the wood structure, which varies in space from the central to the peripheral parts of the stem, and its moisture content, which varies both as a function of the wood structure and as a function of the heating process. The initial moisture content of the wood varies from the center of the tree, through the heartwood and sapwood to the vascular cambium. The following relationship was created to calculate the initial moist density of hardwoods [14], [23], [25]:
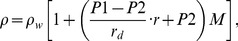
(3)where *ρ_w_* (kg/m^3^) is the density of the dry wood at each location of interest along the radial coordinate, *r_d_* (m) is the radial distance to the vascular cambium, *M* (unitless ratio of water mass per unit dry wood mass) is the maximal moisture content near the bark of the modeled wood section, and the moisture parameters P1, P2 scale the fraction of the maximum inner bark moisture content at the radial locations (a graphic example is shown in Fig. 2 of [1], and see also [23]), and are given in Table 1.

**Figure 2:**
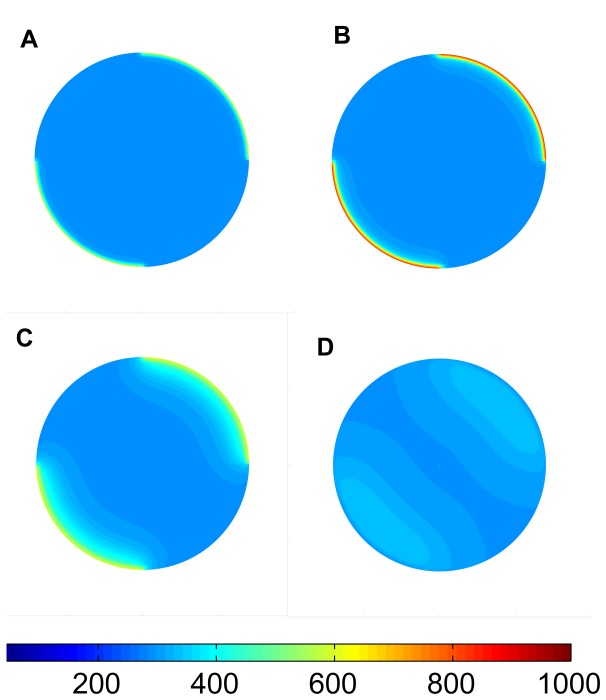
Simulated tree-stem temperature distribution in various time steps. **A.** The heat-up phase (t = 20 sec). **B.** The peak temperature point (t = 80 sec). **C.** The beginning of cool-down phase (t = 100 sec). **D.** After long cool-down period (t = 200 sec). Heating was provided in a heterogeneous way around the virtual stem section, with heat forcing at the upper right and lower left quadrant of the stem and no heat (ambient room temperature) prescribed at the upper left and lower right quadrants. The colors represent temperatures, colorbar is in K.

**Table 1:** Tree species and sections used in the laboratory experiment and parameterization of the thermophysical properties of the species.

Species				Tree #	Section #	Diameter (mm)	Bark thickness (mm)	Mean Moisture Content (%)	Mean Density (g/cm2)
***Acer rubrum*** ** (L.)**				1	1	140	2.84	80.63	0.523
**Parameters**				1	2	131	3.5	80.63	0.523
Wm	P1	P2	P3	2	1	125	3	69.97	0.629
0.8	1	0.5	0.2	2	2	122	3	69.97	0.629
				3	1	136	4	67.89	0.613
				3	2	128	3.9	67.89	0.613
***Acer saccharum*** ** (Marsh.)**				4	1	140	3.7	45.3	0.63
**Parameters**				4	2	120	3	45.3	0.63
Wm	P1	P2	P3	4	3	126	3	45.3	0.63
1	1	0.5	0.2	5	1	128	3	49.21	0.662
				5	2	126	3	49.21	0.662
				6	1	133	3.4	47.14	0.677
***Carya tomentosa*** ** (Lam.)**				7	1	139	8	36.47	0.735
**Parameters**				8	1	151	10.4	36.64	0.69
Wm	P1	P2	P3	8	2	130	8.6	36.64	0.69
0.2	1	0.83	0.26	9	1	140	8.54	38.04	0.731
				9	2	129	6.9	38.04	0.731
***Liriodendron tulipifera*** ** (L.)**				10	1	136	6.2	88.13	0.437
**Parameters**				10	2	132	7	88.13	0.437
Wm	P1	P2	P3	11	1	130	8	87.99	0.401
1	0.63	0.26	0.19	11	2	126	8	87.99	0.401
				12	1	130	8	105.11	0.443
				12	2	126	6	105.11	0.443
***Nyssa sylvatica*** ** (Marsh.)**				13	1	134	6.2	57.12	0.468
**Parameters**				13	2	129	6.1	57.12	0.468
Wm	P1	P2	P3	14	1	132	6	51.82	0.501
0.6	1	0.5	0.2	14	2	102	6	51.82	0.501
				15	1	135	5.8	44.91	0.509
				15	2	110	4	44.91	0.509
***Pinus strobus*** ** (L.)**				16	1	125	3.7	100	0.338
**Parameters**				16	2	105	2.9	100	0.338
Wm	P1	P2	P3	16	3	100	2.4	100	0.338
0.8	0.63	0.26	0.19	17	1	140	3.1	100	0.3
				17	2	140	2.6	100	0.3
				18	1	125	3.1	100	0.323
				18	2	123	3.1	100	0.323
***Quercus prinus*** ** (L.)**				19	1	134	9	40.57	0.633
**Parameters**				19	2	130	7	40.57	0.633
Wm	P1	P2	P3	20	1	120	8	39.39	0.609
0.1	1	0.5	0.2	20	2	114	8	39.39	0.609
				21	1	132	6	40.9	0.631
				21	2	128	8	40.9	0.631
				22	1	124	12	41.97	0.672
				22	2	120	11	41.97	0.672
				22	3	108	8	41.97	0.672
***Quercus rubra*** ** (L.)**				23	1	128	5.9	37.53	0.697
**Parameters**				23	2	128	5	37.53	0.697
Wm	P1	P2	P3	24	1	142	8	35.93	0.724
0.5	1	0.5	0.2	24	2	124	6	35.93	0.724
				25	1	140	9	42.81	0.716
				25	2	132	5	42.81	0.716

*Wm*, water loss rate parameter, estimated per species with the optimization process and properties of all stem sections measured in the lab and simulated by FireStem2D. The moisture parameters P1, P2, and P3 are the fraction of the maximum inner bark moisture content at the radial locations shown in Fig. 2 of [1].

Thermal conductivity was calculated as a function of the wood density and moisture content [26] as:
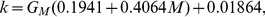
(4)where *G_M_* is specific gravity based on oven-dry mass and volume at moisture content and is equal to *ρ/ρ_w_*,and *M* (unitless ratio of water mass per unit dry wood mass) is the moisture content. Thermal conductivity increases as density, moisture content, temperature, or extractive content of the wood increase.

Heat capacity of the moist wood, *c_p_* (J/kg/K) is calculated as a weighted sum of *c_p0_* (the heat capacity of dry wood), *c_pw_* (the heat capacity of water), and *A_c_* (the energy in the wood-water bond):
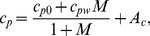
(5)where *c_p0_ = 0.1031+0.00386T; c_pw_* = 3.8+130/(645– *T*), for *T*<630 (K); *c_pw_* = 15, for *T*>630 (K)*;* and *A_c_ = M(−0.06191+2.36*×*10^−4^T-1.33*×*10^−4^)* [27]. The second term in the numerator of the right hand side of the equation is an adjustment for the moisture content of wood, *c_pw_*, and the last term on the right further adjusts the heat capacity to account for the energy in the wood-water bond, *A_c_ = M(−0.06191+2.36·10^−4^T-1.33*×*10^−4^*.

### Thermophysical Properties of Bark

Bark densities are calculated for each control volume based on the dry bark density and the local moisture content, be it inner (live) bark or outer (dead) bark:
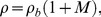
(6)where *ρ_b_* (kg/m^3^) is the density of the dry bark.

Martin [14] provides empirical parameterization for the conductivity of the bark, as a function of the dry density (first term on right hand side), moisture content (second term), and temperature (third term):
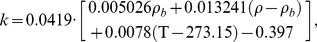
(7)


Martin [14] also provides empirical parameterization for bark heat capacity as a function of temperature and water content:
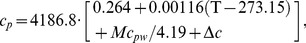
(8)where Δ*_c_* (cal×g^−1^×K^−1^) is an empirical correction for the effect of moisture on heat capacity [14].

### Forcing at the Outer Edge of the Simulated Stem

The simulation is driven by a prescribed forcing of heat flux, which represents the heating that is provided by the fire. This forcing is prescribed by the user as a time series for fire-driven convective and radiant heat fluxes at the outer edge of each numerical section (wedge) around the stem. The model adds this prescribed heat flux to the flux budget at the outer edge of the bark and uses the net flux as a numerical boundary condition, at the outermost node of the bark. The net heat flux at the surface node (representing the outer layer of the bark) is calculated by the model as:
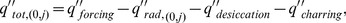
(9)where 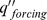 is prescribed by the user in the simulation input file and represents the sum of fire-induced radiant and convective heat fluxes, *q”_rad,(0,j)_* is net radiant heat flux (W/m^2^) exchange with the ambient air, *q” _desiccation_* is heat flux due to desiccation (W/m^2^), and *q” _charring_* is heat flux due to charring (W/m^2^).A simulation typically starts before the fire ignition (or when the fire line is far from the target stem) at which time the initial stem temperature is close to ambient. In that case, the initial desiccation and charring components are zero and do not need to be accounted for as initial conditions.

### Heat Flux Forcing

The users can either prescribe the heat flux from direct measurement or from output of a high resolution fire behavior model that provides the heat flux. Alternatively, users can approximate the values of heat flux based on the fire temperature around the stem. An example for such calculation is provided in the section ‘*virtual experiments for sensitivity analysis*’, below. The air temperature around the stem could be measured in a real fire experiment or generated synthetically using a fire dynamics model or an empirical fire-temperature curve. The total heat flux forcing, 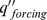, is the sum of a radiant, 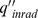, and a convective, 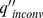, components:
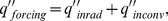
(10)


### Radiant Heat Flux Exchange with the Ambient Air

Radiant heat flux exchange with the ambient air at the outer edge of the simulated stem is prescribed as:
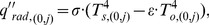
(11)where the right hand side is the black-body radiation, where *ε = 1* is black-body emissivity coefficient, *σ = *5.67×E-8 (W/m^2^K^4^) is the Stefan-Boltzmann constant, *T_s_* is the temperature at the surface node (K) and *T_o_* is the ambient air temperature (*K*) before the fire ignition or far away from the fire. The subscript *(i,j)* marks the coordinate in the polar grid system, where *i* is the grid number from the outer edge along the radial direction and *j* marks the angular wedge number around the stem. Here, *i* = 0 marks a boundary condition at the outer edge of the tree’s bark.

### Desiccation

A major impact of water content in the stem is heat absorption through phase change. The evaporation of water within the bark acts as an additional protective barrier against temperature increases that can damage the stem [23]. Heat flux forcing due to desiccation is characterized as follows:
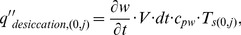
(12)where *V* is control volume (m^3^), and 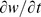 is the time rate of change of the mass of water in a numerical cell of wood, which is solved using the empirical relationship from [28]:
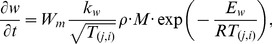
(13)where *W_m_* is a parameter for water loss rate due to high temperature, calculated for the purpose of this paper. Its value was parameterized per species using empirical data from a series of laboratory experiments. *M* is moisture content (unitless ratio), *T* is temperature (K), *ρ* is moist wood density (kg/cm^3^), the coefficient *k_w_* = 6.05×E5 (K^0.5^/s) and the exponential factor *E_w_*/*R* = 5956 (K) are taken from [28]. The empirical parameterization accounts for unmodeled factors, such as resistance to vapor transfer radially through the stem.

### Bark Charring

As the temperature of desiccated bark rises, charring may occur. Charring is the oxidation of the solid carbonaceous material that remains after all moisture and volatile matter has been driven off. Although only a thin portion of the original bark chars, the thermal properties of the charred layer affect the rate of energy transfer into the vital tissues of tree stems [23]. Heat flux forcing due to charring is characterized as follows
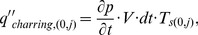
(14)where *V* is control volume (m^3^) and 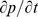 is the time rate of charring. Charring is modeled in a manner analogous to water loss, with the exception that in each time step charring can only occur at the one node that is deeper from a previously charred node. The rate equation is based on [29].
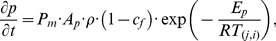
(15)where *P_m_* is the pyrolysis multiplier, *cf* = 0.30 (unitless) is the density fraction, the coefficient *A_p_* = 7×10^7^ (s^−1^) and the exponential factor *E_p_*/*R* = 15,610 (K) are empirical parameters, taken from [29]. The combustion and heat generation by bark material (glowing or flaming combustion) is neglected though it is known to be important for certain species [22], [30]. In our experiments we do not parameterize for *P_m_* as we do not observe charring in any case, because of relatively low temperatures in the bench-scale experiments.

### Tissue Injury

Once the temporal dynamics and spatial distribution of temperature in the stem section is resolved, it is possible to relate the temperature to potential stem injury. Thermally induced tissue viability is described by a temperature-dependent rate equation, where the rate of decline in tissue viability is proportional to current viability [31]–[33]:
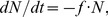
(16)where *N* is the degree of viability for a given node (0 = dead to 1 = alive). The viability progression rate, *f*, is calculated as:
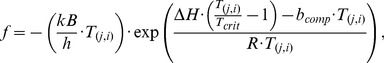
(17)where *T* (K) is temperature at point ( *j,i* ) in the tree stem, *kB* is Boltzman constant, *h* is Planck’s constant and *kB/h* = 2.08×e^10^, *R* = 8.31 (J/mol K) [31] is the universal gas constant, and *ΔH* (kJ/mol) is activation enthalpy, and *T_crit_* and *b_comp_* are parameters of a compensation law relating thermodynamic parameters. Details on parameter estimation are given in Appendix S2.

Viability level can be calculated for a constant temperature exposure by solving equation (17) as a first-order differential equation:
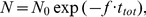
(18)where *N_0_* is initial viability and *t_tot_* is total heating time. If *N* at a certain node falls below 0.001, the node is considered dead and depth of necrosis corresponds to the depth of the most interior dead node after completion of stem cooling. This value of N is arbitrary. The relationship between viability rate and extent of necrosis is exponential and 0.001 represents a 3×log reduction, at which level viability can be assumed negligible as this is a very low value relative to the decimal precision of most of the parameters used in the model (e.g., [34]). Finally, by solving for discrete time intervals, viability level *N* at each node at each time step Δ*t* can be calculated for a temperature regime that varies through time:
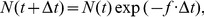
(19)


### Laboratory Experiments

All necessary permits were obtained for the described field studies. Permits were given by the US Department of Agriculture - Forest Service to Matthew B Dickinson and Warren Hellman at the Delaware, OH and Lancing, MI stations of the Forest Service’s Northern Research Station.

Controlled laboratory stem-heating experiments were conducted on stem sections collected from eight tree species during the dormant season. We selected species that are common in eastern and central North America and particularly represent the composition of mixed oak forests in Ohio where the experiment was conducted: *Acer rubrum* (L.) (red maple), *Acer saccharum* (Marsh.) (sugar maple), *Carya tomentosa* (Lam.) (mockernut hickory), *Liriodendron tulipifera* (L.) (yellow-poplar), *Nyssa sylvatica* (Marsh.) *var. sylvatica* (blackgum), *Pinus strobus* (L.) (eastern white pine), *Quercus prinus* (L.) (chestnut oak) and *Quercus rubra* (L.) (northern red oak). Most of these species are typically classified as thin-barked [35], except *Q. prinus* and *C. tomentosa,* which are intermediate (Table 1 includes the details of all stem sections used, including bark thickness). The results of these experiments were used for the parameterization and evaluation of FireStem2D.

The 30 cm stem sections were prepared for heating by first coating the sawn ends with paraffin to reduce water loss during heating. Sections were then wrapped with fiberglass-backed aluminum fire-shelter material (Anchor Industries, Inc., Evansville, IN) containing a 10 cm×10 cm square opening through which bark was heated. The exposed section of each stem was heated using six 25 cm, 400 watt, Type LHP rod heaters (Glo-Quartz, Mentor, OH, USA). Rods were spaced approximately 3 cm apart in an arc positioned 5 cm away from the exposed bark surface. An aluminum shield was placed behind the rods to reflect radiation from the side opposite the target stem. Power to the heating rods was regulated by two variable AC transformers set at 115 VAC.

Prior to heating, stem sections were fitted with three thermocouples (0.52 mm diameter type K probes; Omega Engineering, Inc., Stamford, CT, USA) to monitor temperatures at the bark surface, just beneath the bark surface, and at the cambium layer between bark and wood. Sections were heated until the cambial probe reached a temperature of 343 K, at which time the rods were turned off. In several cases, the cambial probe was inadvertently placed in the wood. Temperatures and heat flux continued to be recorded until the cambial temperature returned to within 10% of the ambient temperature. Total heat flux (convective plus radiative) was measured with a MedTherm Schmidt-Boelter-type heat-flux sensor (Model 64-15T-15R(S)-21210, MedTherm Corporation, Huntsville, AL, USA) logged on a Campbell CR10X micrologger (Campbell Scientific, Logan, UT, USA) at 1-second intervals. The sensor was encased in a 2.5 cm diameter copper cylinder and was positioned on the top of the stem section, flush with the bark surface. Insulating cotton was placed on the top of the stem section to shield the stem from the casing. We examined the MedTherm output for drift associated with temperature rise in the copper cylinder and detected none even over 10 minute exposures. Radiative heat loss likely dampened temperature rise because the copper body was exposed and only received radiation at its front.

MedTherm voltage output was converted to total heat flux (kW m^−2^) through calibration relationships provided by the Rochester Institute of Technology Center for Imaging Sciences (Robert Kremens, Rochester Institute of Technology, unpublished data). The relationship between MedTherm voltage and blackbody heat flux is directly proportional in the range of heat fluxes relevant for this experiment and, consequently, a single 673 K blackbody temperature was used. Total heat fluxes were later adjusted to reflect the estimated total heat flux value at the bark surface at the midpoint of the window. To do this, we used the ratio of two independent MedTherm measurements taken simultaneously during heating regimes identical to those used during the stem heating trials, but with the stem section removed. The MedTherms were placed at the top of the stem section as in the stem heating trials and at the same location as it would have at the surface of the bark at the center of the 10 cm window.

The depth of necrosis into the stem following each stem heating experiment was determined by staining thin stem sections with triphenyl tetrazolium chloride [36]. Measurements taken on three ∼1 cm thick stem cross-sections cut at the midpoint of the window and 1 cm above and below the midpoint were used to calculate average depth of necrosis. Total bark thickness was also measured in all samples and separate outer bark measurements were taken on those species with well-defined inner and outer bark. The depth of the thermocouple probe used to measure the cambial temperature at the midpoint of the window was also determined during the section preparation process. Sections for necrosis and thermocouple depth determination were cut with a power miter saw.

Moisture content and oven-dry density were measured on samples of outer bark, inner bark, and sapwood collected from a section that was removed from the top of each stem just prior to heating. Average moisture content was determined by weighing samples collected from the fresh section and drying the samples at 380±2 K [37]. Oven-dry density (oven dry mass/fresh sample volume) was calculated by weighing another set of samples and coating them in molten paraffin before weighing them again. Sample volume was determined using Archimedes rule by submerging the sample into a beaker of water. The volume of the paraffin (mass/density) was then subtracted to determine the volume of the fresh wood sample. Moisture content was used to estimate oven-dry mass and calculate oven-dry density.

### FireStem2D Simulations of the Laboratory Experiments

We used FireStem2D to simulate the results of the laboratory heating experiments. Simulated stems were divided into 16 uniform angular wedges, and each wedge was discretized into nodes in the radial direction. The distance between nodes in this simulation study was 1 mm. The required inputs to the model were calculated given data from the laboratory experiments and are shown in table 1.

### Model Parameterization

We compared laboratory observations and FireStem2D simulations to estimate a single model parameter: the temperature-driven water loss rate, *W_m_* (eq. 14). Morvan and Dupuy [28] found that its value lies between 0.01 and 1 for a drying fuel particle. We used an optimization process to determine the best approximation to its value. The goal was to find *W_m_* that minimized the error between lab measurements and simulations of necrotic depth and cambium temperatures over all samples of the same species. We define the species-specific error, *E_s_*, as:
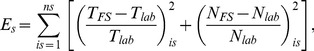
(20)where *ns* is the number of samples of a particular species and the notation *is* represents a counter for sample and corresponding model simulation number. The first term on the right hand side represents model errors in estimation of temperature, *T_FS_* (*K*), is the temperatures calculated with FireStem2D, resampled every 200 seconds, *T_lab_*, the corresponding temperatures measured in the lab. The second term on the right hand side represented model errors in estimation of necrotic depth, *N_FS_* (mm), is the necrotic depth calculated with FireStem2D, *N_lab_*, the corresponding necrotic depth measured in the lab.

### Virtual Experiments for Sensitivity Analysis

To showcase the potential application for the model, we have conducted a series of virtual experiments to test: (1) the effects of the spatial heterogeneity of the distribution of heat around the stem and its interaction with stem diameter, and (2) the effects of height. We compared the predicted extent of stem injury at different heights from surface fire with predictions for a crown fire. We used synthetic forcing, qualitatively based on data from a prescribed surface fire and a crown fire [38].

#### (1) Symmetry experiment

As a fire passes a tree, it usually heats the stem unevenly according to the direction and velocity of the wind. We used FireStem2D to examine the different effects a given total heating dose can have as a function of its distribution over the circumference of a tree. We used a virtual test section with an assumed diameter of 14 cm and the thermal properties of *Pinus strobus* (as measured in the lab experiments, tree 16-1 from Table 1) to evaluate the effects of various spatial distributions of radiant heat-flux forcing and its interaction with stem diameter. The virtual test section was divided into 256 uniform wedges and each wedge was discretized into nodes in the radial direction. The distance between nodes was 0.1 mm. We used the same time series of heat-flux forcing that was applied to tree 16-1 (Table 1) in the lab experiments. We redistributed the heat flux circumferentially in five different ways, varying from uniform to strongly uneven. We further tested two of these five heating cases: an uneven heat-flux distribution (case 1), heating only the windward and leeward sides; and uniform heating around the stem (case 2), for three different assumed diameters of a white pine stem section 16-1 (8 cm, 14 cm and 24 cm).

#### (2) Effect of height above ground

To examine how fire effects change with height, we simulated tree slices in various heights from the ground. This virtual experiment simulated both a surface fire and a crown fire to compare the results. We used virtual *P. strobus* and *Q. prinus* stems with 15 cm diameter and bark thickness of 2 mm and 7 mm, respectively with thermal parameters set as the mean values of sections of tree 16-1 and 19-1, respectively, from the laboratory experiments. The virtual stems were divided in 16 uniform wedges and each wedge was discretized into nodes in the radial direction. The distance between nodes was 1 mm.

#### (A) Surface fire case

We assumed a range of fire temperatures between ambient (pre-fire) and 823 *K*. This range was observed in low-burning prescribed surface fires [39]. We used a normalized, high frequency time series to describe the temporal dynamics of the heat forcing. This time series is based on observations from a prescribed fire conducted from 19–21 March 2011 at the Pine Barrens, NJ, USA (Warren Heilman, U.S. Forest Service, personal communication). Data included air temperature at several heights (1–20 m) above the ground, at 1-minute intervals during the entire day when the fire occurred.

We used air temperature measurements during the fire to calculate the radiant heat flux in different heights (*z*) based on equations 10–[Disp-formula pone.0070110.e017] 12, as follows:
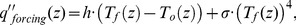
(21)where *T_f_* (z) is the air temperature directly above the fire at height *z* (m) above ground and *T_o_*(z) is the ambient air temperature at the same height before the fire started. Measurements show that *T_f_* (0) = 823 K (temperature at ground level), and *T_f_* (20) = 285 K (temperature above the crown height). We interpolated the heat-flux forcing for each simulated height between these two levels assuming an exponential profile, i.e. 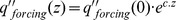, where *c* is a shape parameter that was found by solving this equation for *z* = 20. In this virtual experiment we found that c = −0.0155.

These synthetic forcing conditions were calculated only for sensitivity analysis purposes. Horizontal cross-sections of a *P. strobus* (15 cm diameter, 2 mm bark thickness, and other properties set to those of tree 16.1 in Table 1) and a *Q. prinus* (15 cm diameter, 7 mm bark thickness, and other properties set to those of tree 20-1 in Table 1) stem at different heights were simulated.

#### (B) Crown fire case

To examine the differences between a low-intensity fire and a crown fire and to evaluate how well FireStem2D can depict each, we used data from a crown fire [38] to evaluate the fire effects on a *P. strobus* (15 cm diameter and rest of properties same as tree 16-1 from Table 1) at different heights. The net heat flux is the sum of radiant and convective heat flux. To calculate net heat flux at various heights we used mean values of radiant heat fluxes and temperatures measured at tower 5 located in plot 1 (see Fig. 2 in [38]). All temperature and heat flux data for the surface and the crown fire are gathered in Appendix S3.

## Results

### FireStem2D Parameterization and Evaluation

FireStem2D simulates temperature distribution in a tree stem in two dimensions. Longitudinal energy transport is not simulated. Example temperature distributions during different heating phases are depicted in Figure 2. The images illustrate the radial and circumferential heat transfer due to conduction.

**Figure 3:**
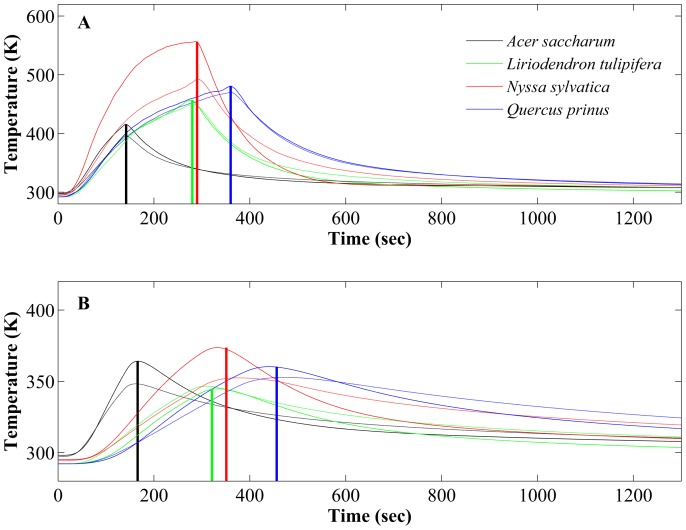
Comparison of laboratory observations and FireStem2D simulations of temperature variation through time for four tree species at different locations in the stem section. **A.** Just beneath surface. **B.** At the cambium. Solid curves mark FireStem2D simulation results and dashed curves mark laboratory observations. Bold vertical lines in each time series marks the observed timing of peak heating, and defines the end of the heat-up phase, and start of the cool-down phase, afterwards. Different color signifies different tree species. Black: *Acer saccharum*; green: *Liriodendron tulipifera*; red: *Nyssa sylvatica*; and blue: *Quercus prinus.* These are examples from the 52 tree sections collected from 25 trees of 8 different species that were tested in the laboratory (Table 1).

**Figure 4:**
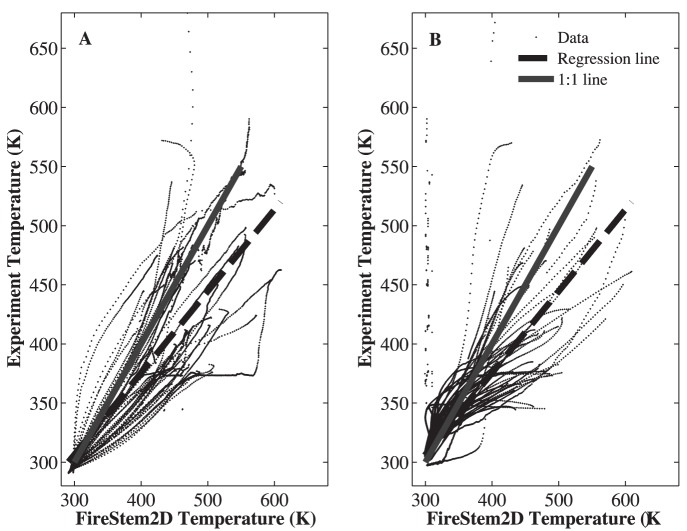
Scatter-plots of measured time series of temperature at a single point (at cambium, at a depth of 1 to 5 cm into the stem) for 52 tree sections collected from 25 trees of 8 different species (table 1). **A.** Heat-up phase (regression line: y = 0.6907(±0.013)x+96(±2.1801), R^2^ = 0.61). **B.** Cool-down phase (regression line: y = 0.6946(±0.0043)x+100.6409(±1.3906), R^2^ = 0.64). Dashed lines mark the overall model-observation fit, solid lines mark the ideal (1∶1) model-observation relationship. FireStem2D predicts the temporal dynamics of temperature in the cambium well, although it slightly overestimates the peak temperatures.

**Figure 5:**
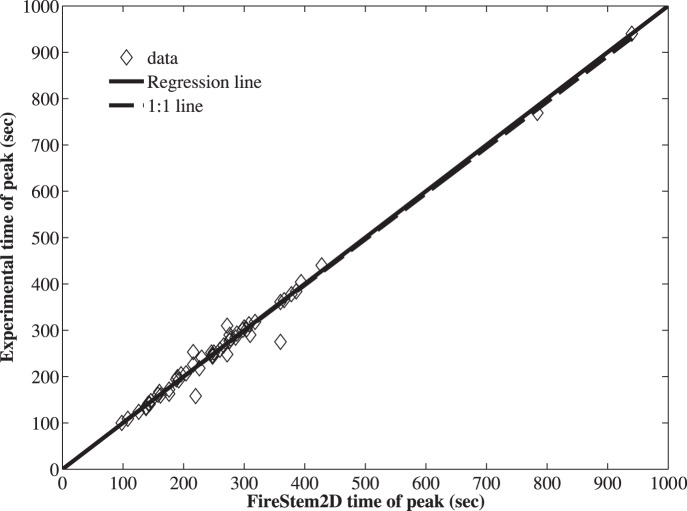
Scatter-plot of FireStem2D simulated versus measured peak-temperature time in all stem sections (Table 1). Dashed line marks the overall model-observation fit, solid line marks the ideal (1∶1) model-observation relationship (regression line: y = 0.9885(±0.0355)x+1.9306(±10.7992), R^2^ = 0.98). The model is not significantly different than the observations.

**Figure 6:**
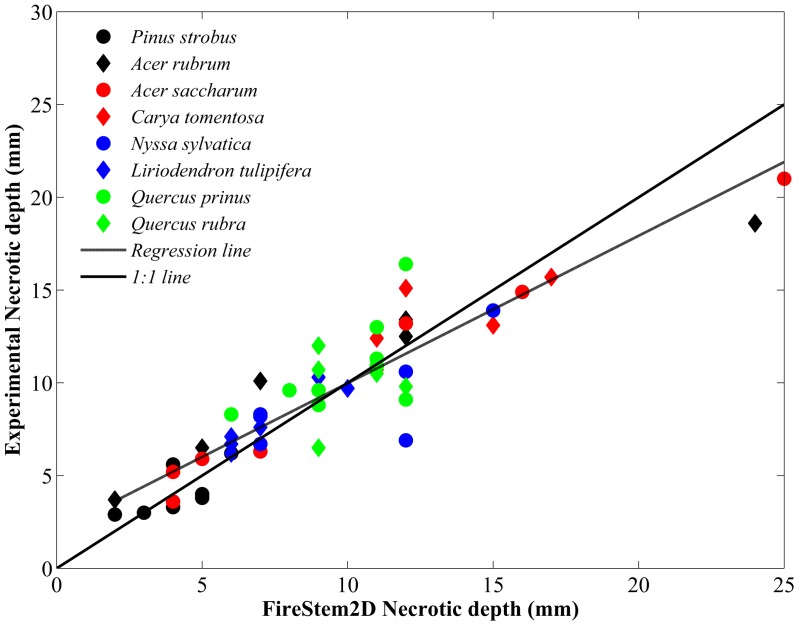
Scatter-plot of necrotic depth of 52 tree sections from 25 trees of 8 different species from the heating experiments (Table 1) compared with the simulated necrotic depth estimated with FireStem2D. Dashed line marks the overall model-observation fit, solid line marks the ideal (1∶1) model-observation relationship (regression line y = 1.051(±0.1325)x−0.6044(±1.3612), R^2^ = 0.84). Regression line and 1∶1 line are not significantly different, proving the very good prediction of necrotic depths.

Results from the physical experiments closely matched results from FireStem2D simulations of the same cases (Figures 3, 4, 5, 6). To illustrate this, Figure 3 presents time series of temperatures of an *Acer saccharum (*tree 4-1), a *Liriodendron tulipifera* (tree 10-1), a *Nyssa sylvatica* (tree 13-1), and a *Quercus prinus* (tree 19-1). The species specific parameter, *W_m,_* showed a wide range between 0.05 in *Carya tomentosa* and *Quercus prinus* to 0.95 in *Acer saccharum* (Table 1).

Figure 4 shows comparisons between the observed time series of temperature at a single point, at a depth of 1–5 cm in 52 tree section from 25 trees of all eight species from the heating experiments, to the simulated temperatures of the corresponding grid-cell with FireStem2D. It is separated into heat-up and cool-down phases. FireStem2D tends to overestimate stem temperature regimes during heating and cooling phases (Fig. 4). However, it provides very good predictions of the time of the peak temperatures for all cases (Fig. 5). FireStem2D also shows very good potential for predicting the necrotic depth of tree stem subjected to heat flux (Fig. 6).

**Figure 7:**
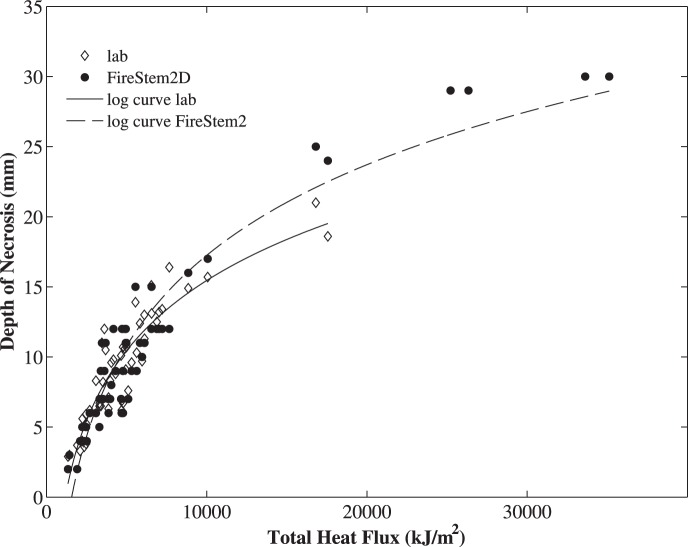
Depth of necrosis (mm) (y-axis) versus the measured total-energy flux integrated over time, THF in kJ/m^2^ (x-axis), for all stem sections used in the lab experiments. Dashed line marks a logarithmic fit of simulation results (y = 21.48(±1.89)log(x)−68.66(±7), R^2^ = 0.93 ), solid line marks a logarithmic fit of observed laboratory results (y = 16.63(±2.1)log(x)−51.05(±7.63), R^2^ = 0.84 ). This model-driven empirical relationship can be used to extrapolate stem heating and vascular cambium necrosis beyond heat fluxes used in experimental stem heating trials.

Equations 17 and 18 imply a direct relationship between temperature variations and necrotic depth. In addition, temperature variations are related to surface heat flux. As a result there is also a direct relationship between surface heat flux and the depth of necrosis. This model-driven empirical relationship can be used to extrapolate stem heating and vascular cambium necrosis beyond heat fluxes used in experimental stem heating trials (Fig. 7).

### Sensitivity Analyses

#### (1) Circumferential heat distribution experiment

**Figure 8:**
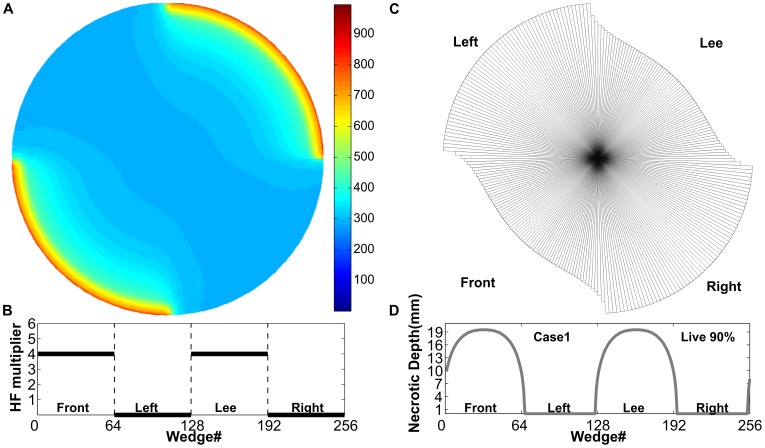
Forcing pattern and results for Case 1. **A.** Temperature distribution in the stem at the peak of heating process. **B.** Schematic illustration of heat forcing that was applied only to the front (upper right quadrant) and lee (lower left quadrant) sides as a plot of heat flux (HF) multiplier vs. the circumferential wedge number. The first wedge is located between 0 and 1.4 degrees from the top (“north”) of the stem and wedge numbers are increased in the clockwise direction. At each wedge, the HF multiplier is applied to the circumferential mean heat-flux forcing. **C.** Remaining uninjured stem after heating. **D.** Necrotic depth of each wedge of the stem.

**Figure 9:**
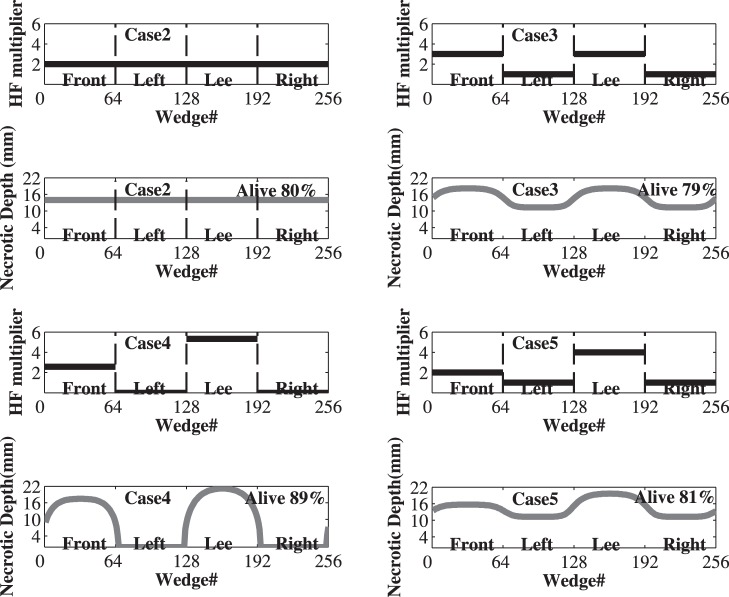
Forcing pattern and necrotic depth results for Cases 2–5. For each case, the upper panel plot shows (black line) how heat forcing was applied around the tree stem and the lower panel for the same case presents the Necrotic Depth (grey line) of each wedge of the stem. Plot layout is the same as panels B and D in figure 8. **Case 2.** Heat forcing was applied evenly around the tree stem. **Case 3.** 3/8 of heat forcing was applied at each of the front and lee side and 1/8 at each of the other sides. **Case 4.** 2.67/8 of heat forcing was applied at front side and 5.33/8 at the lee side. **Case 5.** 2/8 of heat forcing was applied at front side, 4/8 at lee side, and 1/8 at the left and right sides. Our simulation results show that different heating scenarios and heterogeneity of the heat flux around the stem can have different effects on the resulting necrosis and its potential to girdle the tree stem and lead to mortality.

The experiment examines impacts of heat distribution around the tree stem to necrotic depth and its relationship with stem diameter. In our experiment, heat forcing was applied around a virtual *P. strobus* stem (same properties as previously) in five different ways described in Fig. 8 and Fig. 9. The results show that cases 2, 3, and 5, the cases which heat the stem circumferentially with different intensity distributions, have greater effects than cases 1 and 4, which heat only half of the tree’s circumference, at windward and leeward sides. Case 3 (3/8 of total heat at windward and leeward sides, and 1/8 at each of the other sides) has the greatest effects on the tree stem as it leaves only 79% of the tree’s cross-sectional area unaffected by necrosis. However, the results in that case are only slightly different than cases 2 and 5 which leave 80% and 81% unaffected by necrosis, respectively. In this experiment we did not distinguish between sapwood and the total cross-section area. However, the model results can be used to provide specific predictions for the mortality of different critical tissues within the tree. Empirical knowledge of the depth of sapwood relative to the total DBH exists in many species (e.g., [40]) and could be used to predict necrosis of specifically the sapwood. Table 2 also indicates that the greater the diameter of the tree, the larger percentage of stem cross-sectional area survives and the smaller the possibility for tree mortality.

**Table 2:** Percentage of live stem cross-sectional area (percentage of the tree area that remained intact after the burning experiment) for a *Pinus strobus* and a *Quercus prinus* with diameters 8, 14, and 24 cm, for simulation heat case 1 (heating only the front and lee sides of a tree section, along half of the tree’s circumference), and 2 (uniform heating around the full circumference of tree stem).

Tree species	Diameter (cm)	Percentage of live stem area (%)	
		Heat case 1	Heat case 2
*Pinus strobus*	8	31	28
	14	66	64
	24	84	84
*Quercus prinus*	8	46	36
	14	73	71
	24	85	84

#### (2) Impact of height on fire effects

**Figure 10:**
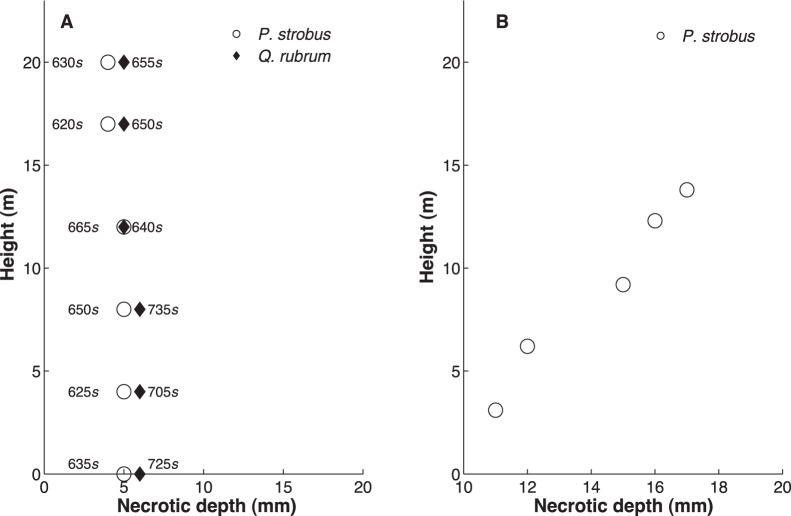
Simulated impact of height (above ground) on necrosis depth in two different virtual experiments. **A.** Low intensity fire: Necrotic depth in different heights: 0 m, 4 m, 8 m, 12 m, 17 m, 20 m for a *P. strobus* (solid markers) and a *Q. prinus* (open markers). The diagram also lists the time at which each necrotic depth was reached. In low intensity fires lower tree levels suffer strongest effects than stem parts higher in the crown. **B.** Crown Fire: Necrotic depth in different heights: 3.1 m, 6.2 m, 9.2 m, 12.3 m, 13.8 m for a *P. strobus*. In crown fires higher tree levels are more strongly affected than the lowers parts of the stem.

(A) Surface fire. In the case of the *P. strobus,* the necrotic depth was almost uniform until a height of 12 m. For the *Q. prinus*, the necrotic depth was deeper than *P. strobus* (6 vs. 5 mm, respectively). It is also noted that in both cases, tree stems reach the greatest necrotic depth in less time at a height of 4 m above the ground (Fig. 10a), at which point the fire temperature and radiative heat flux maximize.

(B) Crown fire. Contrary to the low-intensity fire scenario, the depth of tissue necrosis increases with height for a virtual test tree (Fig. 10b). In our estimation we ignored the fact that bark thickness varies inversely with height [41] to highlight the differences due to the effects of heat forcing.

## Discussion

Results show that FireStem2D accurately predicts temporal variation in stem temperatures (Figs. 3) and necrotic depths (Figs. 6) and the time of peak temperatures in all cases (Fig. 5). However, we assume that the overestimation of the temperatures (Fig. 4) is attributed to the fact that the model’s 2-D structure neglects vertical vapor transport processes. As this is a 2-D model, it does not, by definition, handle three-dimensional processes such as vertical transport of heat and water. In reality, sap is transported upward during the heating, especially when the xylem temperature is very high, as evidenced by bubbling of sap at the top of the stem section, which advects heat and reduces the observed maximal temperature.

Michaletz *et al.* [18] demonstrated that temperature regimes below the bark are affected by xylem water flux through a process that leads to cavitation and vessel deformation. These effects occurred at temperatures ≥338 K, which is higher than the threshold temperature at which tissue necrosis would occur. Although water transport processes are not included in FireStem2D, the model is well suited to provide stem temperature regimes for modeling cavitation and vessel deformation and how it might affect whole-tree heating and injury. Michaletz *et al.* [18] and Butler and Dickinson [42] suggest that a useful next step in tree heating and injury research would be consideration of the effects of heat transport by stem water flux. Significant cooling by water transport is likely to be restricted to actively transpiring trees. Furthermore, we speculate that the conditions under which such cooling is effective will be limited by the deformation and vessel collapse in the stem, because of heat-driven cavitation in foliage and thin branch vessels as a result of extreme vapor pressure deficits in the heat plume during surface fires of even moderate intensity [43]. We predict that cooling by water flux will be most important during low-intensity surface fires that are followed by long-term smoldering and heating at the base of tree stems (e.g., [44]). Incorporating these processes in stem-injury models is the logical next step. However, until more is known about trees’ water relations during fires, it is not clear whether the added complexity and increased simulation time needed to resolve three-dimensional heat and water transport could effectively improve stem-injury prediction accuracy.

The fact that the model is parameterized by species and not by individual tree (same *W_m_* for all sections of same species) contributes to the error between model and observation; however, this error does not lead to a consistent bias. For example, no species is clustered above or below the model prediction line (Fig. 6). The variation in temperature prediction accuracy between trees of the same species indicates that there are some differences in thermal properties between individual trees, due to either phenotypic plasticity or differences in life history. However, an individual-level parameterization would have rendered the model impractical, and the overall good agreement between the model and observation justifies the species-level parameterization.

The sensitivity analysis for the stem and necrotic depth shows the potential application of the model. Fire effects may vary around the circumference of a tree, depending on direction and velocity of the wind. FireStem2D allows the heat from the fire to be prescribed as a dynamic time series and to vary around the stem’s circumference and at different heights.

Tree death is assumed to follow deterministically if the stem is girdled and the tree does not re-sprout. Trees often survive cambial necrosis around part of their circumference. Approximately 15–20% of the cambium is needed to be intact for a tree to survive [20]. Despite the fact that the same cases of heat forcing distributions were used in all the experiments, results varied between the species and life stages, indicating a strong sensitivity to the thermal properties of the bark and wood (Table 2). Specifically, young *Q. prinus* (8 cm) showed roughly 25–50% deeper necrosis than young *P. strobus*. However, for large stems (24 cm) in both species the percentage of live stem did not change with different heating patterns. This indicates that using fire to maintain pine forests by removing young oak trees may be more effective in the earlier stages of forest succession when both pines and oaks are young. FireStem2D can estimate the necrotic depth at any point around the circumference of the tree stem, as well as to estimate what fraction of the total cambium was injured at different heights. This will bridge the knowledge gap between understanding of the fire heat dynamics and predicting of tree stem injury. Therefore, FireStem2D is a useful tool for wildland fire management decision support.

The use of heat-flux boundary conditions makes it possible to couple FireStem2D to a fire behavior model. Fire managers routinely use models to forecast fire behavior [45]. Several models ranging in complexity from the semi-empirical BEHAVE model [46] to the three-dimensional high resolution WFDS [47] and FIRETEC [48] can provide prediction of fire spread rates and intensities when weather conditions and key fuel characteristics are known. These fire behavior predictions can be used to estimate the distribution of heating intensities around a tree bole or stem, which, in turn, serve as inputs to stem injury models such as the Firestem2D model described here [42]. Simpler empirical assumption of fire-line heat could also be used by FireStem2D; however, to really utilize the full advantages of a 2-D model, empirical understanding of the role of wind in the heat distribution around the stem should be incorporated in the heat forcing. FireStem2D can also be used in forecasting the effects of active wildfires if we can predict heat flux accurately, given the fuel conditions in the forest and the climate conditions expected.

FireStem2D could be broadly applied given further parameterization for the thermal parameters of other dominant tree species in different regions. The expanded applicability of FireStem2D makes it a useful tool that can be parameterized for any species and can predict necrotic depths for any species under any heat-flux conditions. In addition, the model provides a strong theoretical basis on which to extrapolate stem heating and vascular cambium necrosis beyond heat fluxes used in experimental stem heating trials (Fig. 8).

## Supporting Information

Appendix S1 FireStem2D: Software and documentation.
(ZIP)

Appendix S2 Thermal tolerance parameter estimation.
(DOCX)

Appendix S3 All temperature and heat flux data for the surface and the crown fire.
(XLSX)
