# Supplementary material for: FireStem2D – A Two-Dimensional Heat Transfer Model for Simulating Tree Stem Injury in Fires
Source: PLoS One. 2013 Jul 19;8(7):e70110. doi: 10.1371/journal.pone.0070110 (PMC3716762; doi:10.1371/journal.pone.0070110)
Supplement: Appendix S1 — FireStem2D: Software and documentation. (ZIP) [file pone.0070110.s001.zip › Appendix1_EfthaliaChatziefstratiou/README.docx]

**Compile the code**

We compiled and ran our code in Linux Redhat, Linux Ubuntu and Mac environments. The suitesparse (v3.5.0) libraries: libumfpack.a, libamd.a, libcholmod.a, libcolamd.a, libufconfig.a (for later versions of suitesparse the ufconfig was renamed to suitesparse_config) were compiled locally and their executables (.a) are stored in prescribed paths. In the makefile, the shared libraries libm 2.5, libblas 3.0.3 (-lm –lblas) were compiled in the system using Redhat RPMs. The libraries (first link) or RPM for Redhat (second link) can be downloaded from the following locations:

suitesparse (libumfpack.a & libamd.a, libcholmod.a, libcolamd.a, libufconfig.a)

[**http://www.cise.ufl.edu/research/sparse/SuiteSparse/**](http://www.cise.ufl.edu/research/sparse/SuiteSparse/)

**http://pkgs.org/centos-6-rhel-6/centos-rhel-x86_64/suitesparse-static-3.4.0-7.el6.x86_64.rpm.html**

libm

[**http://search.cpan.org/dist/Math-Libm/**](http://search.cpan.org/dist/Math-Libm/)

[**http://rpmfind.net//linux/RPM/mageia/cauldron/i586/media/core/release/perl-Math-Libm-1.0.0-3.mga3.i586.html**](http://rpmfind.net/linux/RPM/mageia/cauldron/i586/media/core/release/perl-Math-Libm-1.0.0-3.mga3.i586.html)

libblas

**http://www.netlib.org/blas/**

**http://rpmfind.net/linux/rpm2html/search.php?query=blas&submit=Search+...&system=&arch=**

Please compile the libraries.

The file include.mk lists the compilation variables that will be needed by makefile.

Update the paths to the suitsparse executable in the variable LIBSDir (instead of the current example that lists the paths in our computer).

Update the compiler you want to use in the variable C_COMP. We used the open source GNU compiler g++.

Update the compiler options in C_OPTS. We recommend the optimization option to make it run fast. We used "–O3".

Update the path and name of the source code in SCR. We used "FireStem2D" in the local directory.

Update the name of the resulting executable in EXE. We used "Fire".

Compile the code by using the "Make" command in a linux environment.

You can open and read the code (FireStem2D.cpp) with openoffice.org writer or with any text editor.

Alternatively, you can compile the code on windows or linux environments without using the make file, but will have to link the libraries through the compilation command. We got it working on the same environments as in the example in the include.mk, using:

g++ -O3 -lblas -I/usr/lib -I/usr/lib/include -I/home/chatziefstratiou.1/NewFireStem1/lib -I/home/chatziefstratiou.1/NewFireStem1/lib/suitesparse -o Fire FireStem2D.cpp /home/chatziefstratiou.1/NewFireStem1/lib/suitesparse/libumfpack.a /home/chatziefstratiou.1/NewFireStem1/lib/suitesparse/libamd.a

/home/chatziefstratiou.1/NewFireStem1/lib/suitesparse/libcholmod.a

/home/chatziefstratiou.1/NewFireStem1/lib/suitesparse/libcolamd.a

/home/chatziefstratiou.1/NewFireStem1/lib/suitesparse/libufconfig.a -lm

**Additional instructions for Mac OSX:**

1. Instructions for installing SuiteSparse (v4.2.1) on a Mac are available in the README.txt file of SuiteSparse. Part of that file is reprinted here:

QUICK START FOR LINUX: Just type 'make' in this directory. Then do

'sudo make install' if you want to install the libraries and include files

in /usr/local.

QUICK START FOR MAC: Delete the SuiteSparse_config/SuiteSparse_config.mk

file, and then remove "_Mac" from the *Mac.mk file in that directory. Then

continue as the 'QUICK START FOR LINUX' above.

1. Open a terminal window and navigate to the folder containing Firestem2D.cpp
2. Cut and paste, or type, the following line in the terminal:

g++ -O3 -L /usr/local/lib -lumfpack -lamd -lsuitesparseconfig -lcholmod -lcolamd -lblas -o ExecName FireStem2D.cpp

(Note that you can rename the executable by replacing the word 'ExecName' with whatever name you wish.)

4) The executable should now be available in the same folder.

**Prepare the boundary conditions and input parameters file**

Input file: Separate file that sets the boundary conditions and tree characteristics (we include an example named “Inputfile.bdc”, which was used for one of the simulations in the manuscript Chatziefstratiou et al (2013) Plos One).

The structure of the Inputfile:

“Inputfile” is a .bdc file, “plain text, space separated”.

Row 1: stem radius (m)

Row 2: bark width (m)

Row 3: inner bark width (m)

Row4: radial distance (m) of the points (see fig. 1 of Chatziefstratiou et al (2013) Plos One)

Row 5: initial temperature (C)

Row 6: bark density (Kg/m^3)

Row 7: wood density (Kg/m^3)

Row 8: outer bark initial moisture content

Row 9: inner bark initial moisture content

Row 10: wood initial moisture content

Row 11: number of wedges

Row 12: depth of thermocouple probe (m)

Row 13: total heating time (sec)

Row 14: time step (sec)

Row 15: wmult (see below)

Row 16: pmult (set to 1)

Row 17-End Column1-number of wedges: Heat flux forcing (kj/m^2) at the wedges outer boundary of wedge 1-last wedge. Each row is the forcing during a second of real time, from time 0 till the end of the simulation (total heating time).

Finally we run the program (assuming the executable name is "Fire"):

**./Fire Inputfile speciesnum wmult**

There are two commandline parameters in the execution, speciesnum and wmult. In the table that follows you can find the headers with parameter values. Only the values should be listed in the commandline, not the headers:

speciesnum wmult

Acer rubrum 5 800

Acer sacharum 5 1000

Carya tomentosa 6 200

Liriodendron tulipifera 4 1000

Nysa sa 4 600

Pinus strobus 4 800

Quercus prinus 0 1000

Quercus rubra 0 500

These are the species that we have already parameterized. You can easily parameterize other species (that, however, will require slight modifications to the code). wmult is used as an integer, X1000 times its physical value. The value of wmult in row 14 of Inputfile should be prescribed in physical units (the value in the table above/1000). However, it will be ignored if you are providing a wmult through the commandline upon execution.

**Output files explanation**

**T_Movie.xls** is a space separated text file. It gives snapshots of temperatures for every node. The frequency of the snapshots is determined by the total simulation time/MOVIE_FRAMES. MOVIE_FRAMES is a parameter set in the program in line 26. Line 1 is an empty header line. Line 2 provides the depth in m of the nodes (constant across wedges). The time of the snapshot in seconds is listed in column number 1. In columns 2:end are the temperatures of every node where wedges are in rows and nodes are in columns. Each snapshot is separated by an empty line. Finally, we also submit here a matlab code “fsmovie.m” that creates a movie that shows the temperature change in the stem. The code includes comments that explain the parameters and the commands used.

**Necrosis_Char.xls** is a space separated text file. It gives snapshots of necrotic depth and charring depth in m. Column 1 gives the time. Column 2 gives the necrotic depths. Column 3 gives the charring depth.

**Water.xls** is a space separated text file. It gives snapshots of total water loss and total pyrolized mass in kg. Column 1 gives the time. Column 2 gives the total water loss for every time step. Column 3 gives the total pyrolized mass for every time step.
