# Supplementary material for: FireStem2D – A Two-Dimensional Heat Transfer Model for Simulating Tree Stem Injury in Fires
Source: PLoS One. 2013 Jul 19;8(7):e70110. doi: 10.1371/journal.pone.0070110 (PMC3716762; doi:10.1371/journal.pone.0070110)
Supplement: Appendix S2 — Thermal tolerance parameter estimation. (DOCX) [file pone.0070110.s002.docx]

**Appendix 1**. Thermal tolerance parameter estimation.

Thermodynamic parameters describing pseudo-first-order heat impairment of bark, stem, and foliage were estimated or obtained from three papers. Caldwell [[1](#_ENREF_53)] examined impairment of tissue respiration in discs cut from cucumber leaves. Dickinson and Johnson [[2](#_ENREF_54)] quantified impairment of tissue respiration in samples of live bark. Lorenz [3] estimated survival in populations cortical parenchyma cells sectioned from the stems of seedling. Tissue impairment described by cell death and impairment of cellular respiration are analogous [4]. First-order rate constants are described by the following relationship [5]:

$\text{f=}\frac{\text{k}_{\text{B}}T}{h}\exp\left(\frac{\Delta S}{R}\right)\exp\left(\frac{-\Delta H}{RT}\right)$ , Equation A1

where *f* is the rate constant (*s^-1^*), *k_B_* is the Boltzman constant (1.38 × 10–23 *J K^–1^*), *T* is temperature (*K*), *h* is Planck’s constant (6.63 × 10–34 *J·s*), Δ*S* is the activation entropy (*J mol^–1^ K^–1^*), *R* is the universal gas constant (8.31 *J mol^–1^ K^–1^*), and Δ*H* is the activation enthalpy (*J mol^–1^*). Rate constants for different temperature exposures were estimated from tables reported in [3] and extracted from figures in [1] because raw data were not reported in tables. Thermodynamic parameters estimated from [1,2,3] are shown in Table A1.

We examined whether parameters in Table 1 followed compensation law behavior as did parameters describing heat effects on proteins, viruses, yeasts, and bacteria reported in [5]. Thermodynamic parameters were highly correlated (*R^2^* = 0.997):

$\text{ΔS=}\frac{1}{\text{T}_{crit}}\Delta H-b=\frac{1}{341}\Delta H-258$ , Equation A2

where *T_c_* (*K*) is the compensation temperature and *b* is the intercept (*J mol^–1^ K^–1^*). These values are similar to [5] values, where *T_crit_* ranged from 325-331 and *b* ranged from 268 to 272. The parameter values in Equation A2 and the average of the activation enthalpy estimates in Table A1 (Δ*H* = 318,344) were used in FireStem2D (see Equation 17).

**Table A1.** Thermodynamic parameters estimated by fitting Equation A1 to data. Data from the 25 °C pretreatment group were used from Caldwell (1993). Caldwell tested two varieties of cucumber (Poinsett and Ashley) and mean rate constants were extracted from his Figure 4 so standard deviation estimates were not valid. The temperature range (*T_min_* to *T_max_*) for each experiment is given (ºC).

| **Study** | **Species** | **N** | ***T_min_*** | ***T_max_*** | *ΔS* | Δ*H* |
| --- | --- | --- | --- | --- | --- | --- |
| Dickinson and Johnson (2004) |  |  |  |  |  |  |
| Fall/Winter | *Populus tremuloides* | 17 | 43 | 65 | 372(140) | 217524(46603) |
| Fall/Winter | *Picea glauca* | 11 | 43 | 65 | 455(328) | 246032(107117) |
| Spring/Summer | *Populus tremuloides* | 10 | 43 | 65 | 665(133) | 312522(43889) |
| Spring/Summer | *Picea glauca* | 12 | 53 | 65 | 575(208) | 283968(68354) |
| Spring/Summer | *Pseudotsuga menziesii* | 15 | 43 | 65 | 528(252) | 270036(81790) |
| Spring/Summer | *Pinus contorta* | 17 | 43 | 65 | 518(214) | 267984(70254) |
|  |  |  |  |  |  |  |
| Lorenz (1939) |  |  |  |  |  |  |
|  | *Catalpa speciosa* | 7 | 59 | 66 | 1649(533) | 648885(178796) |
|  | *Pinus strobus* | 11 | 56 | 66 | 895(217) | 396706(72666) |
|  | *Ulmus americana* | 12 | 57 | 68 | 844(244) | 381648(81858) |
|  | *Picea glauca* | 14 | 56 | 69 | 529(176) | 274719(59148) |
|  | *Pinus resinosa* | 9 | 58 | 66 | 872(198) | 389085(66524) |
|  |  |  |  |  |  |  |
| Caldwell (1993) |  |  |  |  |  |  |
|  | *Cucumus sativa - Poinsett* | 10 | 46 | 60 | 87(NA) | 112131(NA) |
|  | *Cucumus sativa - Ashley* | 7 | 40 | 60 | 784(NA) | 337230(NA) |

**LITERATURE CITED**

1. Caldwell CR (1993) Estimation and analysis of cucumber (*Cucumis sativus* L.) leaf cellular heat sensitivity. Plant Physiology 101: 939–945.

2. Dickinson MB, Johnson EA (2004) Temperature-dependent rate models of vascular cambium cell mortality. Canadian Journal of Forest Research 34: 546-559.

3. Lorenz R (1939) High temperature tolerance of forest trees. St. Paul: University of Minnesota Agricultural Experiment Station.

4 Dickinson MB, Jolliff J, Bova AS (2004) Vascular cambium necrosis in forest fires: using hyperbolic temperature regimes to estimate parameters of a tissue-response model. Australian Journal of Botany 52: 757-763.

5. Rosenberg B, kemeny G, Switzer RC, Hamilton TC (1972) Quantitative evidence for protein denaturation as the cause of thermal death. Nature 232: 471-473.
